# Genetic Imbalances in Argentinean Patients with Congenital Conotruncal Heart Defects

**DOI:** 10.3390/genes9090454

**Published:** 2018-09-11

**Authors:** Marisol Delea, Lucía D. Espeche, Carlos D. Bruque, María Paz Bidondo, Lucía S. Massara, Jaen Oliveri, Paloma Brun, Viviana R. Cosentino, Celeste Martinoli, Norma Tolaba, Claudina Picon, María Eugenia Ponce Zaldua, Silvia Ávila, Viviana Gutnisky, Myriam Perez, Lilian Furforo, Noemí D. Buzzalino, Rosa Liascovich, Boris Groisman, Mónica Rittler, Sandra Rozental, Pablo Barbero, Liliana Dain

**Affiliations:** 1 Centro Nacional de Genética Médica, ANLIS, Ciudad Autónoma de Buenos Aires 1425, Argentina; marisoldelea@gmail.com (M.D.); luciadespeche@gmail.com (L.D.E.); bruquecarlos@gmail.com (C.D.B.); mariapazbidondo@gmail.com (M.P.B.); mcperez61@yahoo.com.ar (M.P.); nbuzzalino@gmail.com (N.D.B.); rosaliascovich@hotmail.com (R.L.); bgroisman@gmail.com (B.G.); sandrarozental@yahoo.com.ar (S.R.); pablobarbero63@hotmail.com (P.B.); 2 Instituto de Biología y Medicina Experimental, CONICET, Ciudad Autónoma de Buenos Aires 1428, Argentina; 3 Hospital El Cruce, Dr. Néstor Kirchner, Florencio Varela 1888, Provincia de Buenos Aires, Argentina; sole_massara@hotmail.com (L.S.M.); jaenoliveri@hotmail.com (J.O.); brunpaloma@gmail.com (P.B.); 4 Departamento de Neonatología, Hospital Gandulfo, Lomas de Zamora 1832, Buenos Aires, Argentina; vivicosentino@hotmail.com; 5 Servicio de Genética, Hospital Sor María Ludovica, La Plata 1904, Buenos Aires, Argentina; celestemartinoli@gmail.com; 6 Hospital Dr. Arturo Oñativia, Salta 4400, Salta, Argentina; norma_tolaba@hotmail.com; 7 Hospital Pediátrico Dr. Avelino Castelán, Resistencia 3500, Chaco, Argentina; claupi75@yahoo.com.ar; 8 Servicio de Genética, Hospital Provincial Neuquén “Dr. Eduardo Castro Rendón”, Neuquén 8300, Argentina; eugeniapzaldua@gmail.com (M.E.P.Z.); silvia347@gmail.com (S.Á.); 9 Laboratorio Central de Redes y Programas -MSP, Corrientes 3400, Argentina; vgutnisky@hotmail.com; 10 Hospital Materno Infantil Dr. Ramón Sardá, Ciudad Autónoma de Buenos Aires 1246, Argentina; lilianfurforo@gmail.com (L.F.); rittlerm@gmail.com (M.R.); 11 Departamento de Fisiología, Biología Molecular y Celular, Facultad de Ciencias Exactas y Naturales, Universidad de Buenos Aires, Ciudad Autónoma de Buenos Aires 1428, Argentina

**Keywords:** conotruncal congenital heart defects, 22q11 deletion, copy number variations

## Abstract

Congenital conotruncal heart defects (CCHD) are a subset of serious congenital heart defects (CHD) of the cardiac outflow tracts or great arteries. Its frequency is estimated in 1/1000 live births, accounting for approximately 10–30% of all CHD cases. Chromosomal abnormalities and copy number variants (CNVs) contribute to the disease risk in patients with syndromic and/or non-syndromic forms. Although largely studied in several populations, their frequencies are barely reported for Latin American countries. The aim of this study was to analyze chromosomal abnormalities, 22q11 deletions, and other genomic imbalances in a group of Argentinean patients with CCHD of unknown etiology. A cohort of 219 patients with isolated CCHD or associated with other major anomalies were referred from different provinces of Argentina. Cytogenetic studies, Multiplex-Ligation-Probe-Amplification (MLPA) and fluorescent in situ hybridization (FISH) analysis were performed. No cytogenetic abnormalities were found. 22q11 deletion was found in 23.5% of the patients from our cohort, 66% only had CHD with no other major anomalies. None of the patients with transposition of the great vessels (TGV) carried the 22q11 deletion. Other 4 clinically relevant CNVs were also observed: a distal low copy repeat (LCR)D-E 22q11 duplication, and 17p13.3, 4q35 and *TBX1* deletions. In summary, 25.8% of CCHD patients presented imbalances associated with the disease.

## 1. Introduction

Congenital heart defects (CHD) are a group of structural anomalies of the heart and blood vessels that arise during cardiac embryogenesis and differ in morphology, physiology, and clinical outcome. Congenital heart defects are the most common type of birth defect and one of the major causes of perinatal mortality, with a worldwide prevalence of 1 per 125 births [1,2]. During 2016, the National Network of Congenital Malformations of Argentina (RENAC) detected 352 newborns with critical CHD from a total of 305,452 births, which represents a prevalence of 11.52 affected newborns per 10,000 births [3].

Congenital heart defects include a broad spectrum of malformations that can occur isolated or associated with other malformations. Though CHD pathogenesis is largely unknown, it is widely reported that genetic and non-genetic factors may play an important role [4,5]. Among non-genetic causes, environmental teratogens, maternal exposure to alcohol, thalidomide, seizure medications, infectious agents as rubella, obesity, diabetes mellitus and/or maternal phenylketonuria are recognized as emerging risk factors for CHD [4].

Based on molecular genetic studies, several genes have been identified to be involved in the pathogenesis of CHD, most of which encode cardiac transcription factors such as *NKX2-5*, *TBX5*, *GATA4*, and *GATA6* [5]. In the last years, an increasing number of CHD-associated genes are being identified in humans and in genetically modified mice, including monogenic CHD entities [6,7,8]. Furthermore, it was suggested that oligogenic combinations of inherited genetic variants could explain the majority of CHD that lack a detectable monogenic basis [9,10].

Additionally, chromosomal abnormalities and recurrent copy number variants (CNVs) were also found in a significant number of patients with CHD [11]. The proportion of CHD associated with cytogenetic abnormalities ranges from 9% to 18% [12] and 98% of fetuses with CHD and cytogenetic abnormalities have at least one extracardiac malformation [13]. On the other hand, approximately, 80% of patients with 22q11.2 deletion syndrome (22q11DS) which affects approximately 1 in 4000 live births [14], presented CHD, the majority of them being conotruncal defects (CCHD) [15]. This microdeletion syndrome is mostly caused by a 3 Mb deletion [11,14] which contains approximately 40 genes [16], the *TBX1* and *COMT* being the most relevant ones [17,18]. Other genomic imbalances (microdeletions or microduplications) have also been implicated in CHD, such as 9q34.3 (MIM 610253) and 8p23.1 deletions [19]. These CNVs encompass genes *EHMT1* and *GATA4*, respectively, that when disrupted by a point mutation show a phenotypic spectrum that includes CHD. In addition, a high frequency of CNVs were reported in patients with additional extra cardiac features [7,20]. Moreover, a recent work described an excess of rare CNVs among patients with CCHD in comparison to a healthy population [21].

Conotruncal congenital heart defects are a heterogeneous group of cardiac malformations affecting the outflow tract of the ventricles and the arterial pole of the heart. They represent one of the most prevalent and severe types of CHD and includes, among others, Tetralogy of Fallot (TOF), transposition of the great vessels, and pulmonary atresia with ventricular septal defect. Its frequency is estimated in 1/1000 live births, accounting for approximately 10–30% of all CHD cases [22,23]. In Argentina, the prevalence of CCHD has been estimated in 5.31/10,000 newborns [3]. The presence of chromosomal abnormalities, as well as genomic imbalances in patients with CCHD, are largely studied in several populations; nevertheless, its frequencies are barely published in Latin American countries [24,25].

The aim of this study was to analyze chromosomal abnormalities, 22q11 deletions and other genomic imbalances in a group of Argentinean patients affected with CCHD of unknown etiology. 

## 2. Materials and Methods 

### 2.1. Ethical Approval

All procedures performed in this study were in accordance with institutional and/or national research committee ethical standards and with the 1964 Helsinki declaration and its later amendments or comparable ethical standards. The study was approved by the ethics committee of the Administración Nacional de Laboratorios e Institutos de Salud (ANLIS), Buenos Aires, Argentina (Acta # 14, 16 September 2013). 

Written informed consent was obtained from parents of all patients involved in this study prior to history recording and sampling. 

### 2.2. Subjects

A total of 219 patients (217 unrelated and 2 siblings) under 16 years of age with isolated or associated CCHD was studied. The first cohort was retrospective and consisted of 79 patients referred between May 2013 and May 2014 from four different provinces of Argentina (Chaco, Salta, Neuquén and Buenos Aires). The second cohort was prospective and included 140 patients referred between May 2015 and January 2018 from the city of Buenos Aires and its suburbs. All patients were evaluated by a pediatric cardiologist and a clinical geneticist. A complete physical examination was performed and a detailed individual and familial history were retrieved. Patients with Turner, trisomy 21 or trisomy 18 were excluded from the present study.

The type of CCHD presented by each patient was assorted according to the following classification [26]: TOF, persistent truncus arteriosus (PTA), transposition of the great vessels (TGV), interrupted aortic arch (IAA), pulmonary atresia with ventricular septal defects (PA+VSD), double outlet right ventricle (DORV), and subaortic ventricular septal defect (sVSD). Other major anomalies (MA, defined as birth defects that require significant medical or surgical treatment, have a serious adverse effect on health and development, or a significant cosmetic impact) were recorded according to the definition established in EUROCAT [27], and coded according to the International Classification of Diseases 10th (ICD-10), with the adaptation of the British Association of Pediatrics [28]. 

Of the 219 patients, 130 were males and 89 females (sex ratio 1.46). Their ages ranged from 1 day to 16 years old (mean: 2.43, median: 0.27 years). One hundred and sixty six (76%) patients presented isolated CCHD (iCCHD), and 53 (24%) presented at least another MA (associated CCHD). One hundred and twenty two patients (56%) presented only one CCHD (simple CCHD) and 97 (44%) presented a complex CCHD.

When available, parents were studied to determine if a genomic imbalance was an inherited rearrangement.

All patients presenting MA were subjected to cytogenetic studies. Multiplex Ligation Probe amplification (MLPA) was performed for all patients included in the study while Fluorescence in situ Hybridization (FISH) was performed for those of the first cohort.

### 2.3. Cytogenetic Studies

Chromosome analysis was performed on GTG-banded metaphases (400–550 band resolution) prepared from cultured peripheral blood according to standard protocols.

### 2.4. Fluorescent In Situ Hybridization 

FISH analysis for 22q11 deletion syndrome was performed using probe LSI DiGeorge/VCFS region (Tuple1) (Vysis, Downers Groove, IL, USA). Hybridization to metaphase slides was performed according to the manufacturer’s recommendations.

### 2.5. Multiplex Ligation Probe Amplification Analysis

DNA was extracted from peripheral blood lymphocytes by standard procedures. Multiplex Ligation Probe Amplification analysis was performed using SALSA P250-B1 MLPA kit and SALSA P424B2 MLPA kit (MRC-Holland, Amsterdam, The Netherlands) according to the manufacturer’s recommendations and including four control samples in each reaction. The P250-MLPA kit is the “high density” 22q11 probemix that contains 48 MLPA probes for several regions, including 4q35, 8p23, 9q34, 10p14 (DGS2), 17p13, 22q11 and 22q13. The P424-MLPA kit includes 37 genomic regions previously associated with CHD (e.g.,: 1q21, 2p22, 2q37, 7q22, 10q25, 17p11, 22q11) based on a study by Sørensen et al. [11]. Capillary electrophoresis was performed using ABI 3500 Genetic Analyzer (Applied Biosystems, Foster City, CA, USA), along with the GeneScan 600 LIZ Size Standard (Applied Biosystem, Foster City, CA, USA). For data analysis, the application Coffalyser.net (MRC-Holland Amsterdam, The Netherlands) was used.

### 2.6. TBX1 Exon 7 Sequencing

DNA was amplified with primers TBX1F: 5’-ctagggaacccgctctgttc-3’ and TBX1R: 5’-ccggccctacctttctcc-3’ encompassing exon 7 and intronic splicing sites. Purification of PCR products was performed using AccuPrep PCR Purification (Bioneer Corporation, Daejeon, Korea) kit according to the manufacturer’s recommendations and sequenced using the BigDye Terminator Cycle Sequencing reaction kit v1.1. Capillary electrophoresis was performed using ABI 3500 Genetic Analyzer (Applied Biosystems, Foster City, CA, USA)

### 2.7. Statistical Analysis

Contingency tables were accomplished to determine if there were significant differences in gender and in different CCHD types among all CCHD patients and the latter also among patients presenting 22q11 deletion, as well as in the frequency of 22q11 deletion among the two cohorts analyzed. For the latest analyses, the results obtained in the MLPA were taken into account considering only those patients presenting the typical 3 Mb and the shorter 1.5 Mb deletion. Statistical analysis was performed using Chi^2^ test or Fisher test with GraphPad version 5.0 for Windows (GraphPad Software, La Jolla, CA, USA, www.graphpad.com). Confidence intervals at 95% for binomial distribution proportion were calculated using STATA software (StataCorp. 2017. Stata Statistical Software: Release 15. College Station, TX: StataCorp LLC, https://www.stata.com/).

## 3. Results

### 3.1. Description of the Cohort

Two hundred and nineteen patients who fulfilled the inclusion criteria were referred to ascertain the genetic causes of CCHD. Sex ratio was 1.46 (males/females 130/89), being statistically significant (*p* < 0.001).

Table 1 depicts the distribution of different types of CCHD among patients, gender, and if they are simple or complex CCHD, isolated or associated. Tetralogy of Fallot was the most frequent CCHD (86 patients) followed by TGV (44 patients). 

### 3.2. Cytogenetic and Fluorescent In Situ Hybridization Analyses 

A total of 47 out of 50 patients with MA were successfully karyotyped and all of them had a normal chromosome complement. FISH analysis was successfully performed in 61 out of the 79 samples from the first cohort. Thirteen (21.3%) patients presented the 22q11 deletion. Results obtained for this group of samples by FISH and MLPA techniques were fully concordant. Thus, only MLPA analysis was performed in the second cohort. 

### 3.3. Multiplex Ligation Probe Amplification Analysis

From the 219 samples included, 217 were successfully analyzed using the P250-B1 MLPA kit, (76 from the first cohort and 141 from the second). We found a frequency of 22q11 deletion of 0.21 (16/76) for the first cohort, while for the second one, the frequency was 0.25 (35/141). Considering that this difference was not statistically significant (*p* = 0.53), both cohorts were described and analyzed together.

**Figure 1:**
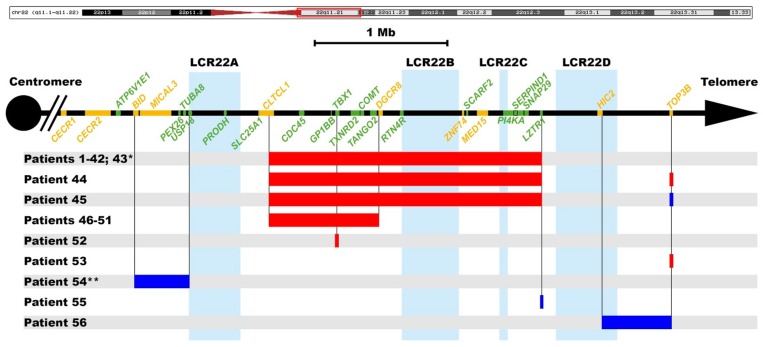
Microdeletions and microduplications in the 22q11 region among patients with CCHD. The red rectangle in the ideogram above displays the chromosome 22q11 region included in the analyses. Horizontal red bars indicate deletions, while the blue ones represent duplications. The OMIM genes are in green. Regions involving the different low copy repeats (LCRs) are highlighted in light blue. The imbalances extensions are delimited by vertical black lines. When only one probe from the MLPA kit is deleted or duplicated, only one vertical line is displayed. * This patient also presented a 9q34.3 duplication involving only one probe. ** This patient also presented a deletion involving at least 406 Kb in 4q35.12.

A total of 63 patients presented at least one chromosomal imbalance: 51 (23.5%) had 22q11 deletion, 45 (20.7%) of them comprised the typical 3 Mb deletion and 6 (2.8%) had the shorter 1.5 Mb deletion (Figure 1). Three of the patients having the typical 22q11 deletion also presented another chromosomal imbalance, one of them a 9q34.3 duplication involving only one probe in chr9:139805146-139805210 (hg18), the second one a deletion and the third a duplication in *TOP3B*, respectively.

From the remaining, 12 patients (5.5%) out of 63 in whom a chromosomal imbalance was detected with the P250-B1 MLPA kit, 5 presented different imbalances also in chromosome 22, 3 a duplication and 2 a deletion (Figure 1). In one of them (patient #54), the duplication was located in the distal region between the LCR D-E, and in another one in the Cat-eye syndrome (CES) region upstream of the LCR A. This last patient also presented a deletion involving at least 406 Kb in 4q35.12 (chr4:186303263-187390398, hg18). In the remaining patient, the duplication involved only one probe in the distal region between LCR C-D (*LZTR1*). Among deletions, 1 patient presented a deletion of one probe (*TOP3B*) in the LCR D-E region, and the last one presented a deletion in the exon 7 of *TBX1* gene. The presence of a putative genetic variant that could prevent the probe hybridization was excluded after sequencing this exon and neighboring intronic regions (Supplementary Figure S1).

Lastly, seven patients presented imbalances in different chromosomes (Table 2). Five patients presented imbalances in 17p13.3: two of them a deletion and three a duplication. In addition, one patient presented duplication in 8p23 and another patient in 9q34.3. 

The presence of other putative CHD imbalances was further screened using a second MLPA kit (P424B2) in a subset of 66 samples among the 154 remaining patients. Another three patients (4.5%) presented a chromosomal imbalance, being in all of them a duplication of only one probe in each three different genes: *TNFRSF4*, *ACVR2A* and *ACTC1* (Table 2). Therefore, a total of 66 patients with different CCHD types presented at least one chromosomal imbalance. Clinical characteristics of the patients in whom imbalances have been found are summarized in Supplementary Table S1. 

In 12 CCHD patients, samples from family members were available to analyze if the imbalance was inherited. Eight out of nine 22q11 deletion patients were de novo and only one was inherited from the mother. She disclosed short stature (10th percentile), short palpebral fissures, small ears with hypoplastic lobes, long nose with bulbous tip and broad columella; high palate with bifid uvula, nasal voice, small hands with slender fingers, history of CHD (VSD) and mild to moderate intellectual disability. In addition, the three imbalances in *TOP3B* were inherited from the patients’ healthy mothers. Finally, one of the duplication in 17q13.3 in *GEMIN4* was inherited from the healthy father.

### 3.4. Analysis of the Distribution of 22q11 and Other Imbalances among Different Groups of CCHD Patients

**Figure 2:**
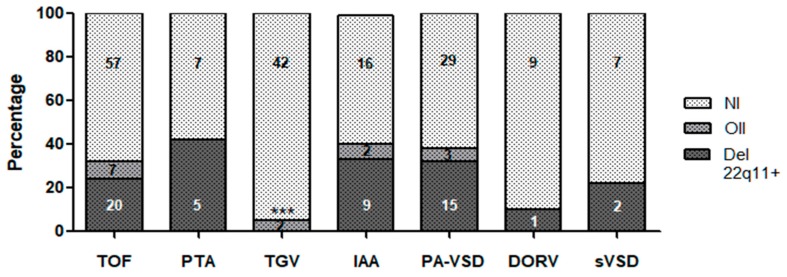
Distribution of 22q11 deletion and other imbalances in the different groups of CCHD studied. Del 22q11 refers to the 3 Mb and 1.5 Mb deletions. CCHD: Conotruncal congenital heart defect; TOF: Tetralogy of Fallot; PTA: Persistent Truncus Arteriosus; TGV: Transposition of the Great Vessels; IAA: Interrupted Aortic Arch; PA + VSD: Pulmonary Atresia with Ventricular Septal Defect; DORV: Double Outlet Right Ventricle; sVSD: Subaortic Ventricular Septal Defect; OII: Other isolated imbalances: refers to imbalances found in patients excluding those observed also with the 22q11 deletion; NI: No imbalances. Inside each chart, the number of patients in each category. Patients who had more than one CHD were included in both categories.

Figure 2 displays the distribution of 22q11 deletion and other imbalances, considering the different groups of CCHD (otherwise indicated, from now on, the 3 Mb and the 1.5 Mb deletions are grouped when a 22q11 deletion is mentioned). 

As shown, the 22q11 deletion was observed in 34% of the patients presenting PA + DVS, in 33% with PTA, in 30.8% with IAA, and in 16.6% presenting TOF. Conversely, just one out of ten patients with DORV presented the 22q11 deletion, while none of the 44 patients presenting TGV had this deletion. Only the absence of the 22q11 deletion observed in patients with TGV was statistically significant (*p* < 0.001, Supplementary Table S2). Patients with TOF and IAA were the most prevalent among those who showed another isolated imbalance (8.4% and 7.7%, respectively) (Figure 2 and Supplementary Table S1).

**Figure 3:**
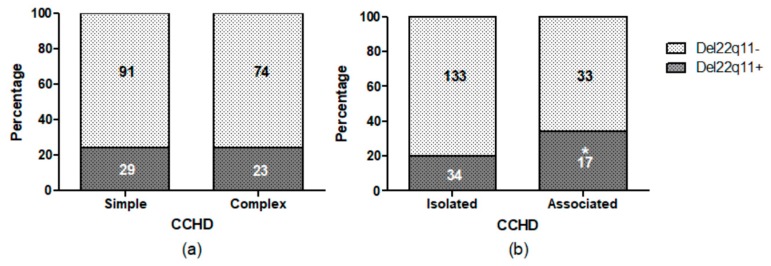
Distribution of the 22q11 deletion in simple or complex CCHD (**a**) or in CCHD presented as isolated or associated (**b**). Del 22q11 refers to the 3 Mb and 1.5 Mb deletions. CCHD: Conotruncal congenital heart defects. Inside the charts, numbers of patients in each category. * *p* < 0.05.

Figure 3 shows the distribution of 22q11 deletion among patients presenting or not another CCHD (complex or simple CCHD, respectively), as well as those presented as isolated or associated. No statistical significance was found when the distribution between simple or complex CCHD was analyzed. Nevertheless, 22q11 deletion was more frequently associated to CCHD with at least another MA when compared with cases of iCCHD (*p* < 0.05). Among the 17 patients with 22q11 deletion and MA, 5 presented TOF, 5 PA + VSD, 5 IAA, and 2 PTA. In 13 of them, the MA observed is described in the 22q11DS (Supplementary Table S1). From the total of 51 children who had the 22q11 deletion, 34 presented CHD without any other MA: 15 presented TOF, 3 PTA, 2 IAA, 11 PA + VSD, 1 DORV and 2 IAA and sVSD (Supplementary Table S1).

## 4. Discussion

Congenital heart defects are the most common birth defects and the leading cause of mortality in the first year of life [29]. Conotruncal defects are a subset of serious and relatively common CHD, defined as defects of the cardiac outflow tracts of great arteries. Previous studies have established the important role of genetic causes as risk factors in the pathogenesis of CCHD. Chromosomal abnormalities and CNVs contribute to disease risk in patients with syndromic and/or non-syndromic forms of the disease [20,30]. In addition, several studies have found that sequence variants in genes disrupted by CNVs, like *TBX1*, *EMHT1* and *ELN* among others, can also be implicated in the pathogenesis of these diseases [7,31,32]. 

In the present study, patients from different provinces of Argentina presenting CCHD were referred to try to ascertain genetic causes of the defect with unknown etiology.

When compiling the patients’ data, the proportion of males exceed that of females, in accordance with previous reports [3,33]. Moreover, as was previously reported, patients with TGV accounted for the biggest difference in this proportion [34].

It is worth noting that in our sample, TOF was the most prevalent CCHD exceeding twice the patients with TGV and PA + VSD that were the most prevalent after TOF. This distribution differs from the prevalence observed in some registries like RENAC [3] and EUROCAT [27], in which patients with TGV and TOF were the most frequently found and in relative similar proportions. The main reason for this difference might rely on the fact that our study is not representative of the prevalence of CCHD in newborns, since it was conducted by pediatric referrals. Indeed, the distribution observed may reflect a bias of a possible greater referral of patients with TOF due to its more widespread association with 22q11 deletion. 

In order to ascertain the genetic causes of the disease, the first approach was to analyze the presence of chromosomal abnormalities in patients with associated CCHD. Using conventional GTG-banded metaphases, we were unable to evidence any cytogenetic abnormality among the samples analyzed. Although chromosomal abnormalities have been reported in approximately 12% of patients with CHD [12], this percentage accounts mostly for patients presenting trisomy 21 or trisomy 18 syndromes that were excluded in the present study. 

In addition, we analyzed imbalances in the 22q11 region as well as in other CNVs previously found to be associated with CCHD. The deletion in 22q11 was studied in the first cohort of 79 patients using two different techniques, FISH and MLPA. Fluorescent in situ Hybridisation could evidence a putative chromosomal rearrangement, but only the typical 3 Mb deletion could be revealed using the aforementioned probe. Conversely, MLPA analysis also evidenced duplications and deletions encompassing different sizes besides the typical one of 3 Mb. Multiplex Ligation Probe Amplification also allows the interrogation of additional regions associated with CHD in a single experiment, however, devoid of elucidating putative chromosomal rearrangements. From the total of 79 patients, only 77% were successfully analyzed by FISH, being the results fully concordant with those of MLPA analysis. Taking into account these results and considering that the FISH technique is expensive and time-consuming, only MLPA was used for the second cohort studied. 

It is well known that the 22q11 Deletion Syndrome, also known as Velocardiofacial (VCF)/DiGeorge syndrome, is the most common microdeletion syndrome in humans and that CCHD are one of the most common phenotypic manifestations. However, it should be noted that the 22q11 deletion was also found in a significant number of patients with isolated CCHD [35,36,37]. 

In our cohort, the proportion of CCHD patients having 22q11 deletion was in accordance with previous reports of Goldmuntz et al. [38] and Barisić et al. [39]. However, other studies reported lower frequencies ranging from 2.5% to 6.8% of 22q11 deletion in liveborns [24,30,40] and from 4.7% to 7.1% in fetuses [23,41,42]. Differences among studies, including ours, could be due to the proportion of the CCHD types analyzed, some of which are most frequently associated to the presence of the 22q11 deletion, like TOF or PTA, or less frequently associated like TGV or DORV. In addition, it should be noted that a higher frequency of 22q11 deletion has been described among Hispanics compared with Caucasians, African Americans, and Asians [43]. 

Among the different CCHD, IAA, PTA and TOF showed the highest frequencies of 22q11 deletion similar to studies in other populations [38]. Of note, these CCHD are also the most prevalent among patients with 22q11DS [44]. Nevertheless, we also found a remarkable high frequency of 22q11 deletion among patients with PA+VSD. On the other hand, Zhang et al. assessed TOF and PA + VSD as the most frequent CCHD in patients with the deletion [44]. 

From our cohort, none of the 44 patients with TGV presented a 22q11 deletion. Although some reports published describing patients with TGV and 22q11 deletion [15,45,46], it is well known that this association should be considered as sporadic [44]. Indeed Ryan et al. [47] and Marino et al. [15] found that around 1% (or even less) of patients with a 22q11 deletion disclosed TGV. 

When analyzing the presence of other cardiac anomalies in our patients, we found no differences in the frequencies of 22q11 deletion patients, although it was significantly higher in those who presented at least another extracardiac major anomaly. Despite this, one of the major results from our study is that among the 51 patients presenting the deletion, 34 disclosed isolated CCHD. These results highlight the importance of genetic screening of patients with conotruncal heart defects even in the absence of other anomalies that could suggest the presence of the deletion. Patients with 22q11DS may not have all the major features, but the mere presence of a CCHD may guide this diagnosis. Moreover, early diagnosis of 22q11 deletion could be of great importance to aid in the management of putative late complications, immunodeficiency, hypocalcemia, developmental and speech delay, behavioral phenotypes, and psychiatric illness [48]. 

Lastly, although a small proportion of family members were available for genetic studies, most of the 22q11 deletions were de novo, with only around 10% of the cases being inherited, similar to findings reported in previous studies [49].

Excluding the 3 Mb and 1.5 Mb deletions in the 22q11 region, 14 different types of microdeletions or microduplications were also found in the analyzed samples. Nevertheless, only four may be considered clinically relevant: a distal duplication in the LCR D-E in the 22q11 region, a 1 Mb deletion in 17p13.3, a deletion in 4q35, and a deletion in *TBX1*. 

The duplication in 22q11 between the LCR D-E was found in one patient with PA + VSD, short philtrum, broad nose, micrognathia, and hypocalcemia. Similar microduplications encompassing this region have been previously reported associated with variable phenotypes, including iCCHD [50,51,52]. 

The 17p13.3 deletion found in one of the patients with TOF encompasses at least 1 Mb in a more telomeric region of a well-characterized chromosome deletion syndrome (OMIM: 247200) in which approximately 20% of the patients present TOF, PTA, or septal defects [53]. Although it was suggested that deletions or point mutation of *PAFAH1B1* are sufficient enough to cause lissencephaly [53], a most severe phenotype compromising facial dysmorphisms, growth restriction and cardiac defects, among others, are due to a contiguous deletion of a region, including at least the three genes *PAFAH1B1*, *CRK* and *YWHAE* [54]. Due to limitations of the MLPA technique, we cannot determine if the imbalance also comprises the *CRK* and *PAFAH1B1,* since it is located closer to a centromeric region than the last probe that the kit has in this region. Smaller microdeletions involving *YWHAE* but distal to *PAFAH1B1* have been reported by other authors (see [53,54]), disclosing distinct phenotypes of mild intellectual disability, moderate to severe growth restriction, white matter abnormalities, and developmental defects in brain and eye. Thus, for this patient, performing an array comparative genomic hybridization (array-CGH) could help to define the length of the imbalance and the genomic regions involved.

Deletion in 4q35 has been reported in patients with CCHD. Eugen-Matthias Strehle et al. [55] studied 20 patients with 4q deletions and found significant genotype-phenotype correlations at a single gene level, linking specific phenotypes to individual genes. The authors reported an imbalance slightly smaller than ours and proposed a potentially critical region for 4q35 associated to CHD compromising the *SORBS2* gene (616349) [56]. In addition, the patient from our cohort disclosed also a duplication on the CES region. This duplicated region is mapped to 22q11.21, but neither overlaps with the VCF/DiGeorge locus nor includes the *CECR1* and *CECR2* genes, the major candidate genes for heart/facial and neurologic/eye features of the CES. Considering that the patient presented only a CCHD (TOF), we hypothesized that her phenotype is caused by the deletion in chromosome 4. Nevertheless, we cannot rule out that the duplication in chromosome 22 is contributing to some extent with the patient’s phenotype, or alternatively, that this duplicated region could contribute to the development of additional clinical signs in the future. 

Finally, one patient presented an intragenic deletion in *TBX1*. Different size of deletions in *TBX1* have been reported in patients with VCF/DiGeorge syndrome [57,58]. In addition, *TBX1* point mutations have also been linked to non-syndromic CHD in humans, including isolated TOF, VSD, PA, DORV, IAA and aortic arch anomalies [59,60,61,62]. Moreover, some authors reported that *Tbx1* deficiency in mice may cause dosage-dependent anomalies of the cardiac outflow tract [63,64,65].

Additionally, three patients had imbalances in the *TOP3B* region, all of them inherited from their healthy mother: two presented a deletion (one patient also carried the typical 3 Mb 22q11 deletion) and one a duplication. Although imbalances in *TOP3B* are assumed as benign CNVs, it should be noted that some authors reported that microdeletions/duplications of this locus were more frequently found in CHD patients than in controls [40]. More patients should be studied along with healthy individuals to determine if this locus represents a genetic risk factor for CCHD in our population. 

Most of the remaining imbalances found in the patients were duplications involving only one probe in which the extent of the duplication could not be ascertained. Although array-CGH would contribute to defining the chromosomal region involved and its putative implication in disease development, in general these loci were associated with CHD when a deletion was present [19,53,66,67,68].

## 5. Conclusions

This is the first report studying chromosomal abnormalities and CNVs in a large cohort of CCHD patients from Argentina and one of the few reports from Latin America. Our study found that 23.5% of CHD patients from this cohort had the 22q11 deletion. The distribution among the different types of CCHD was similar to those reported in other populations. 22q11 deletion was more frequent among patients with associated CCHD. Nevertheless, the fact that 34 out of 51 of the 22q11 deletion patients presented only CHD, highlights the importance of testing the deletion in patients presenting a conotruncal heart defect. Moreover, four clinically relevant imbalances, different from the 22q11 deletion, were also observed among the patients studied: a distal LCR D-E 22q11 duplication, and a 17p13.3, a 4q35 and *TBX1* deletions. In summary, 25.8% of CCHD patients presented a causative CNV.

Although we found other microduplications and microdeletions, additional studies should be necessary in order to elucidate if they represent genetic risk factors for the development of the syndrome.

Accurate diagnosis of the genetic causes of CCHD is important for a proper clinical follow-up, in order to assist in the prevention of morbidity and reduction of mortality in these patients.

## Figures and Tables

**Table 1:** Distribution of different CCHD types by gender, presence of other CHD and major extracardiac anomalies among patients.

CCHD	*n*	Gender		Presence of Other CHD		Presence of Extracardiac MA	
		F	M	Simple	Complex	Isolated	Associated
**TOF**	86	36	50	84	2	69	17
**PTA**	12	5	7	8	4	7	5
**TGV**	44	15	29	21	23	40	4
**IAA**	27	10	17	20	7	16	11
**PA + VSD**	48	21	26	35	13	30	18
**DORV**	10	4	6	2	8	6	4
**sVSD**	8	5	3	5	3	6	2

CCHD: Conotruncal congenital heart defect; TOF: Tetralogy of Fallot; PTA: Persistent Truncus Arteriosus; TGV: Transposition of the Great Vessels; IAA: Interrupted Aortic Arch; PA + VSD: Pulmonary Atresia with Ventricular Septal Defect; DORV: Double Outlet Right Ventricle; sVSD: Subaortic Ventricular Septal Defect; F: Female; M: Male; MA: Major/s Anomalies; Simple: Only one CCHD; Complex: with another CHD. Note that the total number of CCHD is greater than 219 since some patients had more than one CCHD: IAA + TGV (*n* = 1); IAA + sVSD (*n* = 1); DORV + sVSD (*n* = 2); TGV + PA + VSD (*n* = 3); TGV + DORV (*n* = 2); TGV + DORV + PA + VSD (*n* = 1); DORV + TOF (*n* = 1); PA + sVSD (*n* = 1); IAA + PTA (*n* = 3).

**Table 2:** CCHD patients with imbalances out off the 22q11 region *.

Subject ID	Gender	Cytoband	Chromosome Region (hg18)	Event	Probes	OMIM Genes
**57**	M	17p13.3	Chr17:169259-1211325	Del	4	*VPS53 GEMIN4 BHLHA9 YWHAE*
**58**	M	17p13.3	Chr17:1211255-1211325	Del	1	*YWHAE*
**59**	M	17p13.3	Chr17:1211255-1211325	Dup	1	*YWHAE*
**60**	M	17p13.3	Chr17:596607-596671	Dup	1	*GEMIN4*
**61**	M	17p13.3	Chr17:596607-596671	Dup	1	*GEMIN4*
**62**	M	8p23	Chr8:11653542-11653609	Dup	1	*GATA4*
**63**	M	9q34.3	Chr9:139731001-139731062	Dup	1	*EHMT1 b*
**64**	F	01p36.33	Chr1:1137299-1137363	Dup	1	*TNFRSF4*
**65**	M	02q22.3	Chr2:148401174-148401249	Dup	1	*ACVR2A*
**66**	M	15q14	Chr15:32872975-32873043	Dup	1	*ACTC1*

* Does not include patients having imbalances in different chromosomes disclosed in Figure 1. CCHD: Conotruncal congenital heart defects; M: Male; F: Female; Del: Deletion; Dup: Duplication.

## Supplementary Materials

The following are available online at http://www.mdpi.com/2073-4425/9/9/454/s1, Figure S1: Representative partial electropherogram of exon 7 of the *TBX1* gene from patient # 52; Table S1: Clinical characteristics of the patients in whom imbalances have been found. Table S2: Frequency of the 22q11 deletion among different types of CCHD.
