# Supplementary material for: Genetic Imbalances in Argentinean Patients with Congenital Conotruncal Heart Defects
Source: Genes (Basel). 2018 Sep 11;9(9):454. doi: 10.3390/genes9090454 (PMC6162499; doi:10.3390/genes9090454)
Supplement: Supplementary file 1 [file genes-09-00454-s001.pdf]

GENETIC IMBALANCES IN ARGENTINEAN PATIENTS WITH CONGENITAL CONOTRUNCAL HEART DEFECTS

Marisol Delea[1], Lucía D. Espeche[1§]; Carlos D. Bruque[1,2 §]; Maria Paz Bidondo[1] ; Lucía S. Massara[3], Jaen Oliveri[3], Paloma Brun[3], Viviana R. Cosentino[4], Celeste Martinoli[5], Norma Tolaba[6], Claudina Picon[7], Maria Eugenia Ponce Zaldua[8], Silvia Ávila[8] ; Viviana Gutnitzky[9], Myriam Perez[1] ; Lilian Furforo[10], Noemí D. Buzzalino[1], Rosa Liascovich[1], Boris Groisman[1], Mónica Rittler[10], Sandra Rozental[1], Pablo Barbero[1] and Liliana Dain[1,,11*]

SUPPLEMENTARY MATERIAL

Figure S1. Representative partial electropherogram of exon 7 of the *TBX1* gene from patient # 52.

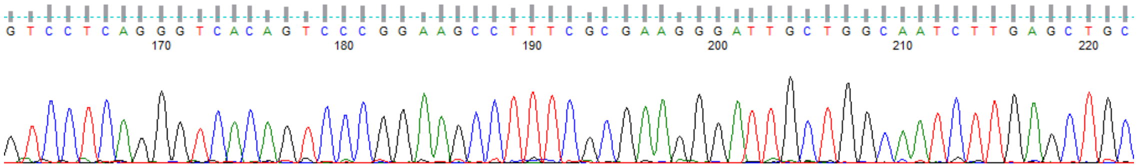

The sequence shown corresponds to the MLPA P250 kit *TBX1* probe 10810-L14347 hybridization region.

Table S1 Phenotype and chromosomal imbalances found in CCHD patients

Patient	Gender	CHD	Additional clinical features	Cytoband	Chromosome region (hg18)	Event	Probes (n)
**Typical 22q11.21 deletion**							
**1**	M	TOF	Dysplasic ear and hooding of the upper lids	22q11.21	chr22:17621597 -19679261	Del	14
**2**	M	IAA	Ocular hypertelorism and dysplasic ear, small thymus	22q11.21	chr22:17621597 -19679261	Del	14
**3**	M	IAA	Blepharophymosis, epicanthal folds, bulbous nose, dysplastic ears, cleft palate, long fingers, umbilical hernia, hypocalcemia, hypothyroidism and neurodevelopmental delay	22q11.21	chr22:17621597 -19679261	Del	14

Patient	Gender	CHD	Additional clinical features	Cytoband	Chromosome region (hg18)	Event	Probes (n)
**4**	M	IAA	Pierre Robin sequence (submucosal cleft palate, glossoptosis, retrognathia), blepharophymosis, straight and short bridge nose, short and marked filtrum, fine and long fingers	22q11.21	chr22:17621597 -19679261	Del	14
**5**	M	PA + VSD	Thymus agenesis, dysplastic ears, bulbous nose	22q11.21	chr22:17621597 -19679261	Del	14
**6**	M	PA + VSD	Dysplastic ears	22q11.21	chr22:17621597 -19679261	Del	14
**7**	M	PTA		22q11.21	chr22:17621597 -19679261	Del	14
**8**	F	PA + VSD		22q11.21	chr22:17621597 -19679261	Del	14
**9**	M	TOF	Complete cleft palate	22q11.21	chr22:17621597 -19679261	Del	14
**10**	F	TOF	Complete cleft palate	22q11.21	chr22:17621597 -19679261	Del	14
**11**	M	PA + VSD	Unilateral renal hypoplasia	22q11.21	chr22:17621597 -19679261	Del	14
**12**	M	PA + VSD	Developmental delay,velopharyngeal incompetence, hemihypoplastic tongue	22q11.21	chr22:17621597 -19679261	Del	14
**13**	F	TOF		22q11.21	chr22:17621597 -19679261	Del	14
**14**	M	PA + VSD		22q11.21	chr22:17621597 -19679261	Del	14
**15**	M	IAA + sVSD		22q11.21	chr22:17621597 -19679261	Del	14
**16**	F	PTA + AVSD		22q11.21	chr22:17621597 -19679261	Del	14
**17**	F	PA+VSD + Hypoplasic pulmonary branches	Cleft lip	22q11.21	chr22:17621597 -19679261	Del	14
**18**	M	TOF		22q11.21	chr22:17621597 -19679261	Del	14
**19**	F	PA + VSD	Unilateral microtia, pilonidal sinus	22q11.21	chr22:17621597 -19679261	Del	14
**20**	F	PTA	Proptosis, absence of eyebrows, microstomia, retrognathia, long thumbs. developmental delay, seazures, feeding problems, rectovesical fistula, Unilateral lower limb reduction	22q11.21	chr22:17621597 -19679261	Del	14

Patient	Gender	CHD	Additional clinical features	Cytoband	Chromosome region (hg18)	Event	Probes (n)
**21**	F	PA + VSD		22q11.21	chr22:17621597-19679261	Del	14
**22**	M	IAA + VSD		22q11.21	chr22:17621597-19679261	Del	14
**23**	F	PA + VSD	Blepharophymosis, bulbous nose, microstomia, long and thin toes	22q11.21	chr22:17621597-19679261	Del	14
**24**	M	TOF		22q11.21	chr22:17621597-19679261	Del	14
**25**	F	TOF	Thymus agenesis	22q11.21	chr22:17621597-19679261	Del	14
**26**	M	TOF	Developmental delay, blepharophymosis with upslanting palpebral fissure, wide nose, small mouth, dysplastic ears, short neck and tapering fingers.	22q11.21	chr22:17621597-19679261	Del	14
**27**	M	TOF	Blepharofimosis, displasic ears, nose with prominent nasal root and bulbous nasal tip, microstomia and speech delay	22q11.21	chr22:17621597-19679261	Del	14
**28**	F	TOF	Hypocalcemia and immune deficiency	22q11.21	chr22:17621597-19679261	Del	14
**29**	F	IAA	Broad nose, misshapen ears, microstomia, micrognathia and feeding problems	22q11.21	chr22:17621597-19679261	Del	14
**30**	F	PA + VSD	Feeding problems and hypocalcemia	22q11.21	chr22:17621597-19679261	Del	14
**31**	M	sVSD	Hypocalcemia	22q11.21	chr22:17621597-19679261	Del	14
**32**	M	TOF	Plagiocephaly, blepharophimosis, retrognathia, misshapen ears, long and thin fingers	22q11.21	chr22:17621597-19679261	Del	14
**33**	M	TOF	Cryptorchidism and microcephaly	22q11.21	chr22:17621597-19679261	Del	14
**34**	F	TOF		22q11.21	chr22:17621597-19679261	Del	14
**35**	M	TOF		22q11.21	chr22:17621597-19679261	Del	14
**36**	M	TOF	Dysplastic ears, broad nose with square tip, micrognathia, feeding disorders and developmental delay	22q11.21	chr22:17621597-19679261	Del	14
**37**	F	TOF	Enophtalmia, hypocalcemia and seizures	22q11.21	chr22:17621597-19679261	Del	14

Patient	Gender	CHD	Additional clinical features	Cytoband	Chromosome region (hg18)	Event	Probes (n)
**38**	M	TOF	Plagiocephalia, blepharofimosis, retrognatia, rotated low set ears, long and thin fingers	22q11.21	chr22:17621597 -19679261	Del	14
**39**	M	TOF		22q11.21	chr22:17621597 -19679261	Del	14
**40**	F	TOF		22q11.21	chr22:17621597 -19679261	Del	14
**41**	F	PA + VSD	Blepharophymosis, mirognathia, dysplastic ears, short philtum , board nose and microstomia	22q11.21	chr22:17621597 -19679261	Del	14
**42**	M	PTA	Dysplastic ears, thing fingers, 46, XX male genitals	22q11.21	chr22:17621597 -19679261	Del	14
**Typical 22q11.21 deletion with additional chromosomal imbalances**							
**43**	M	PTA	Dysplasic ears	22q11.21	chr22:17621597 -19679261	Del	14
				9q34.3	chr9:139805146 -139805210	Dup	1
**44**	M	COA + IAA + VSD		22q11.21	chr22:17621597 -19679261	Del	14
				22q11.22	chr22:20652996 -20653065	Del	1
**45**	F	IAA + sVSD + ASD + Bicuspid aorta		22q11.21	chr22:17621597 -19679261	Del	14
				22q11.22	chr22:20652996 -20653065	Dup	1
**Short 22q11 deletion**							
**46**	F	PA + VSD + ASD + PDA	Developmental delay	22q11.21	Chr22:1762159 8-18453678	Del	9
**47**	F	IAA	Clubfoot	22q11.21	Chr22:1762159 8-18453678	Del	9
**48**	F	TOF		22q11.21	Chr22:1762159 8-18453678	Del	9
**49**	M	TOF		22q11.21	Chr22:1762159 8-18453678	Del	9
**50**	M	PA + VSD	Thymus agenesis	22q11.21	Chr22:1762159 8-18453678	Del	9
**51**	M	DORV	Blepharophimosis, broad nose, dysmorphic ears, microstomia, feeding problems and development delay	22q11.21	Chr22:1762159 8-18453678	Del	9
**Other chromosomal imbalances in 22q11**							
**52**	F	TGV + VSD		22q11.21	chr22:18133286 -18133352	Del	1

Patient	Gender	CHD	Additional clinical features	Cytoband	Chromosome region (hg18)	Event	Probes (n)
**53**	F	PA + VSD		22q11.22	chr22:20652996 -20653065	Del	1
**54**	F	TOF		22q11.21	chr22:16606684 -17012985	dup	3
**54**	F	TOF		4q35.1-2	chr4:186303263 -187390398	del	2
**55**	F	PA + VSD		22q11.21	chr22:19679191 -19679261	Dup	1
**56**	M	PA + VSD	Feeding problems, short filtum, broad nose, micrognathia and hypocalcemia	22q11.2	Chr22:2012944 2-20653065	Dup	3
**Other isolated chromosomal imbalances**							
**57**	M	TOF	Blepharophymosis, broad nose	17p13.3	chr17:169259- 1211325	Del	4
**58**	M	TOF		17p13.3	chr17:596607- 596671	Dup	1
**59**	M	TOF		17q13.3	chr17:596607- 596671	Dup	1
**60**	M	TOF		17p13.3	chr17:1211255- 1211325	Del	1
**61**	M	IAA		17p13.3	chr17:1211255- 1211325	Dup	1
**62**	M	TGV		8p23	chr8:11653542- 11653609	Dup	1
**63**	M	TOF	Developmental delay, axial hypotonia	9q34.3	chr9:139731001 -139731l062	Dup	1
**64**	F	IAA + hypoplasic LV		1p36.33	chr1:1137299- 1137363	Dup	1
**65**	M	TOF	Learning disability, Postaxial polydactyly and dysplasic ear	02q22.3	chr2:148401174 -148401249	Dup	1
**66**	M	TGV + VSD		15q14	chr15:32872975 -32873043	Dup	1

CCHD: Conotruncal congenital heart defect, **TOF:** Tetralogy of Fallot, **PTA:** Persistent Truncus Arteriosus, **TGV:** Transposition of the Great Vessels, **IAA**: Interrupted Aortic Arch, **PA+VSD**: Pulmonary Atresia with Ventricular Septal Defect, **DORV**: Double Outlet Right Ventricle; **sVSD**: subaortic Ventricular Septal Defect. **ASD:** Auricular Septal Defect. **PDA**: Patent Ductus Arteriosus. **AVSD**: Atrioventricular Septal Defect. **LV**: Left Ventricle. **F**: Female, **M**: Male. **Dup**: duplication **Del:** deletion

Table S2: Frequency of the 22q11 deletion among different types of CCHD.

CCHD	N	del22q +	Frequency	CI		p
**TOF**	84	20	0.24	0.15	0.34	1
**PTA**	12	5	0.42	0.15	0.72	0.667
**TGV**	44	0	0	0	0.08	0,001
**IAA**	27	9	0.33	0.17	0.54	0,544
**PA + VSD**	47	15	0.32	0.19	0.47	0.49
**DORV**	10	1	0.1	0	0.45	1
**sVSD**	9	2	0.22	0,03	0.6	1

CCHD: Conotruncal congenital heart disease, **TOF**:Tetralogy of Fallot, **PTA**: Persistent Truncus Arteriosus, **TGV:** Transposition of the Great Vessels, **IAA:** Irrupted Aortic Arch, **PA+VSD**: Pulmonary Atresia with Ventricular Septal Defect, **DORV:** Double Outlet Right Ventricle; **sVSD**: subaortic Ventricular Septal Defect. Note that the total number of CCHD is greater than 217since some patients had more than one CCHD: IAA+TGV (n=1); IAA+sVSD (n=1); DORV+sVSD (n=2); TGV+PA-VSD (n=3); TGV+DORV (n=2);TGV+DORV+PA-VSD (n=1), DORV+TOF (n=1); PA+sVSD (n=1); IAA+PTA (n=3). **22q11+:** Presence of a 3 Mb or 1.5 Mb 22q11 deletions. **CI:** Confidential intervals.
